# Identification of Compassion Fatigue Risk Profiles in Veterinarians: Implications for Prevention and Professional Well-Being

**DOI:** 10.3390/ejihpe15100217

**Published:** 2025-10-21

**Authors:** David Cobos Sanchiz, José María León-Pérez, Francisco Javier Cantero-Sánchez, José María León-Rubio

**Affiliations:** 1 Faculty of Social Sciences, Department of Education of Social Psychology, Pablo de Olavide University, 41013 Seville, Spain; 2 Carmides Research Laboratory, Faculty of Psychology, Department of Social Psychology, University of Seville, Ramón y Cajal Campus, 41018 Seville, Spain; leonperez@us.es (J.M.L.-P.); fcantero@us.es (F.J.C.-S.); jmleon@us.es (J.M.L.-R.)

**Keywords:** compassion fatigue, veterinarians, emotional profiles, cluster analysis, emotional regulation, veterinary communication

## Abstract

Compassion fatigue is a widely recognized phenomenon in human care settings, but it has been little explored in the veterinary field, despite sharing many of the same determinants. This study aimed to (1) identify distinct emotional risk profiles in veterinarians based on their levels of compassion fatigue and satisfaction; (2) estimate the relative prevalence of compassion fatigue in each of these profiles; and (3) analyze the predictive value of sociodemographic variables (gender, age, cohabitation) on belonging to these profiles. A cross-sectional study was conducted with 135 practising veterinarians. An abbreviated version of the ProQOL scale, adapted to the animal context, was used. Its two-dimensional structure (compassion fatigue and satisfaction) was validated using confirmatory factor analysis. Hierarchical cluster and k-means analyses were performed on the factor scores, which identified four emotional profiles: (1) intense emotional involvement, (2) emotional detachment, (3) functional distancing, and (4) high emotional risk. The latter grouped 23% of the sample, while 50.4% presented significant levels of emotional exhaustion. Finally, an ordinal regression was applied, which showed that being over 44 years of age (OR = 2.11) and living with a partner (OR = 1.94) increase perceived emotional risk, with no significant effects of gender. The findings highlight the need for training initiatives that enhance emotional regulation and communication with animal guardians or owners, while promoting sustainable, ethically responsible, and emotionally healthy professional practice.

## 1. Introduction

### 1.1. Conceptualization and Risks Associated with Compassion Fatigue

Compassion fatigue (CF) is understood as the emotional cost of caregiving derived from sustained empathic involvement with others’ pain and traumatic experiences. Figley (1995) initially defined it as a progressive emotional, physical, and spiritual exhaustion syndrome affecting healthcare and social service professionals. He equated it with secondary traumatic stress (STS), whose symptoms—intrusive memories, avoidance, hypervigilance, or sleep disturbances—are similar to those of post-traumatic stress disorder, although mediated by the empathetic experience of another’s trauma. Figley and Roop (2006) described CF as a mental and emotional exhaustion process resulting from the constant pressure to empathetically and effectively respond to the care demands of animals and owners. This reaffirms the vicarious trauma. With the introduction of the ProQOL scale, Stamm (2005) operationalized CF as STS and, later, in an expanded formulation, defined it as the negative dimension of professional quality of life, composed of two interrelated elements: burnout (BO), which reflects a gradual deterioration marked by exhaustion and loss of effectiveness, and STS, which expresses the more sudden impact of vicarious trauma (Stamm, 2005, 2010). CF is a central construct for understanding the psychosocial risks associated with caregiving in both human and animal care.

Despite considerable progress in research on CF in human health and care professions, its study in animal care remains scarce (Andrukonis et al., 2020; González-Ramírez & Landero-Hernández, 2025). However, the need to analyze CF in the veterinary community is justified by their exposure to unique risk factors inherent to the profession. This is linked to the specificity of the bond these professionals establish with animals and the emotional demands derived from the relationship with their owners. Likewise, the high prevalence of this condition and associated mental health problems reinforce the relevance of its study (Chan & Wong, 2023; Hill et al., 2020; Schlanser et al., 2021).

Key risk factors include frequent exposure to euthanasia and the need to provide support to both the animal and its caregivers (Cameron et al., 2025; Deponti et al., 2023); ongoing contact with animals that have suffered abuse or neglect (Musetti et al., 2020; Williams et al., 2022); and managing clients in contexts of loss or conflict (Dow et al., 2019). Added to this is the ethical tension or moral distress derived from balancing animal welfare with the owners’ economic and emotional limitations. These circumstances can force decisions that are not the most advisable, such as delaying diagnostic tests or opting for treatments with limited effectiveness (Kogan & Rishniw, 2023; Kogan et al., 2020).

Other factors that increase vulnerability to CF have also been identified, such as adverse working conditions (high workloads, staff shortages, low pay, or negative work environments) and certain individual characteristics, including high empathic sensitivity, lack of personal resources for self-care, or the presence of previous mental health problems (Malone, 2025).

Due to their breadth and interaction, a professional culture that discourages emotional expression and a lack of training in dealing with highly emotionally charged situations exacerbate the complexity of the risks exposed, which increases isolation and limits coping strategies (Matte et al., 2019; Morales Foster & Maples, 2014). Hence, there is a need to raise awareness of CF in the veterinary clinical setting and to promote both personal resources and organizational initiatives that foster safe environments and support among colleagues (Marshman et al., 2021; Sosnicki & Reynolds, 2024).

### 1.2. Prevalence of Compassion Fatigue Among Veterinarians

Raising awareness of CF requires recognizing its presence and assessing its magnitude by studying its prevalence, which is key to understanding its real impact and guiding prevention and intervention strategies with certainty.

Few studies have explicitly estimated the prevalence of CF in the veterinary field despite its relevance. Most studies do so indirectly within the framework of broader research on the mental health and quality of life of these professionals. However, the available evidence gives us an idea of its impact and challenges for future research.

Most studies in North America have documented a high prevalence of chronic fatigue syndrome and mental health problems. In Canada, Best et al. (2020) and Perret et al. (2020) applied the ProQOL scale and found STS rates of 69.8% and 65.4%, respectively, as well as 41.7% BO and 31.6% CS. Perret et al. (2020) reported a prevalence of suicidal ideation of 26.2%, which is much higher than that of the general population.

In the United States, Schlanser et al. (2021) evaluated military veterinarians and found that none of them had high levels of BO or STS, while more than half (52%) scored high on CS. The authors attribute these results to specific characteristics of military personnel, such as specialized training, institutional cohesion, and resilience and psychological support programs. Similarly, in a study conducted by Young et al. (2024) with technicians and veterinarians working with laboratory animals, none of the participants scored high on BO or STS. Moreover, 35% of the patients reported high CS, suggesting a more resilient profile than in other veterinary groups. In contrast, Pavan et al. (2020) reported that 81.6% of laboratory facility staff had symptoms consistent with CF and BO. In addition, Hill et al. (2020) showed that 87% of animal care workers exceeded the STS cutoff point, while Figley and Roop (2006), Randall et al. (2021), Thurston et al. (2021), and Wolf et al. (2024) confirmed equivalent results in veterinarians, technicians, and animal shelter staff, although in the latter study, 53.5% also scored high on BO. Finally, Kramper et al. (2023) demonstrated that 3.6–13.9% of veterinarians met PTSD criteria, depending on how traumatic events were defined; the number of exposures to highly stressful events was significantly associated with suicidal ideation, depression, substance abuse, and low work well-being.

In South America, González-Ramírez and Landero-Hernández (2025) reported that, in a sample of 438 veterinarians, 82.2% and 67.1% had high CF and high BO, respectively, although smaller-scale national studies, such as those in Chile and Ecuador, reported lower prevalences (23.5% and 17%, respectively), highlighting internal disparities similar to those observed in North America.

The evidence is more limited in other contexts, illustrating the diversity of scenarios. Chan and Wong (2023) found that 66.1% of veterinarians in Hong Kong had moderate STS levels, and only 3.6% reported high BO. The authors attribute this apparent paradox—a highly competitive market coupled with low levels of burnout—to a cultural style that inhibits the open expression of negative emotions and. In Australia, Rohlf et al. (2022) observed that 23.5% and 24.4% of veterinarians had high STS and BO levels, respectively. Although these figures are lower than those reported in South America and North America, they are clinically relevant to occupational health.

In Europe, the direct prevalence findings are limited. At the continental level, Jansen et al. (2024) conducted two surveys of more than 25,000 veterinarians and found that 22–23% required sick leave due to mental health problems in 2018 and 2023, whereas the scores on the Warwick-Edinburgh Mental Wellbeing Scale remained low. This study identified women and young professionals as the most vulnerable groups in all European countries. In Spain, Macía et al. (2022), with a sample of 602 veterinarians, reported 4.3% with high STS and 2% with high BO, along with 20% with elevated CS; they also highlighted a tendency to resort to psychotherapy, anxiolytics, and risky levels of alcohol consumption. For their part, Goñi-Balentziaga and Azkona (2024) identified differential risk profiles associated with the type of animal and rates above the national average in psychotherapy (16.3%) and use of psychotropic drugs (18.8%) with 80 professionals from biomedical research centers. On a more clinical level, San Martín González et al. (2023) found that 23% of Spanish veterinarians presented burnout, according to the Maslach Burnout Inventory (MBI), with a higher prevalence in small animal clinicians (75.3%) and women (66.1%). Hernández-Esteve et al. (2024) reported 36.9% exhaustion and 33.3% cynicism among veterinarians in the Canary Islands. Overall, the Spanish data reflect lower prevalences of CB than in other countries, but BO and psychological distress indicators are comparable to those in Europe.

In summary, the prevalence of CB in the field of animal care shows marked geographical and occupational variability, with figures ranging from moderate values in some European countries to rates above 80% in South America or in specific sectors in North America. In this comparative framework, Spain has lower percentages of STS and BO—around 2–4% in large samples—but with rates of 23–36% burnout in certain studies and indirect indicators of psychological distress (psychotherapy, use of psychotropic drugs, alcohol consumption) that suggest a considerable impact on the mental health of the profession. This paradox suggests that, although CF may manifest itself with less intensity than in other contexts, the overall well-being of Spanish veterinarians is equally a cause for concern and requires specific preventive measures. The discrepancies between countries are not only due to methodological and cultural differences but also to the weight of sociodemographic variables—such as age, gender, or cohabitation with a partner—which seem to play a role as significant predictors of perceived emotional risk and whose consideration is essential to advance in the identification of CF risk profiles.

### 1.3. Sociodemographic Variables and Risk Profiles

Sociodemographic characteristics, which have been linked to a higher or lower risk of CF in various studies, may help explain the variability in prevalence. Several studies have suggested that female veterinarians report higher BO and STS levels, although these associations are usually limited to the descriptive level and do not always reach statistical significance in predictive models (Best et al., 2020; Wolf et al., 2024; Young et al., 2024). Other studies have found no significant relationships between gender and CF (Perret et al., 2020; Rohlf et al., 2022; Schlanser et al., 2021). However, differences in emotional expression, caregiving roles, or levels of empathy could explain women’s perceived greater emotional burden in professional practice (Dow et al., 2019).

Regarding age, several studies have reported that younger professionals tend to have higher levels of BO and STS and lower CS than their older colleagues (Best et al., 2020; Dow et al., 2019; Pavan et al., 2020; Perret et al., 2020). This inverse relationship could be attributed to less experience in dealing with animal suffering or a possible self-selection effect, as those who cannot tolerate the emotional burden tend to leave the profession. Some studies have observed a weak positive association between older age and a lower risk of CF (Andrukonis et al., 2020), while others have found no statistically significant links (Rohlf et al., 2022; Macía et al., 2022).

Finally, few studies have suggested that sharing one’s life with another person could have a protective effect against general psychological distress (Reif-Stice et al., 2023), although not necessarily against EB (Dow et al., 2019; Goñi-Balentziaga & Azkona, 2024).

Taken together, these results indicate that the predictive value of sociodemographic variables is relative and depends on their interaction with other personal and contextual factors, which seem to offer a greater explanatory capacity for emotional distress in veterinarians (Rohlf et al., 2022; Young et al., 2024). This justifies the need for analytical approaches that transcend bivariate associations and allow for the identification of differential risk and protection profiles. Exploring configurations based on combined scores of compassion fatigue and compassion satisfaction is particularly relevant, given that experiencing both dimensions simultaneously is common (Figley, 2002; Wolf et al., 2024). Although evidence in the veterinary field is still limited, this perspective has shown relevant results in other healthcare contexts (Lee et al., 2016; Yaghmour et al., 2024).

Techniques such as cluster analysis, latent class models, and semantic networks offer valuable tools for identifying personal trajectories—some of them paradoxical—that conventional models tend to obscure, and which would allow for the design of prevention and support interventions better adapted to the reality of veterinarians.

### 1.4. Objectives

This study adopts an exploratory and descriptive approach based on three main objectives. First, to identify compassion fatigue risk profiles through a cluster analysis of compassion fatigue and satisfaction scores on the ProQOL scale. Second, to estimate the relative prevalence of compassion fatigue based on these profiles. Third, to analyze whether sociodemographic variables—gender, age, and cohabitation with a partner—predict belonging to these profiles

Achieving these objectives may contribute to consolidating the conceptualization of CF in the veterinary field by providing empirical evidence on its structure, prevalence, and correlates. On a practical level, identifying risk profiles, in addition to raising awareness of a problem that is often ignored in the veterinary profession, could facilitate the design of preventive programs that are better adapted to the specific needs of the identified profiles. This would, in turn, facilitate the prioritization of measures to be taken, as well as a more efficient allocation of preventive resources.

Overall, this study is a first step toward building a more conscious professional culture among those who care for the emotional well-being of other living beings. Recognizing this psychological strain will not only promote the well-being of professionals but also foster a more ethical, compassionate, and sustainable veterinary practice.

## 2. Materials and Methods

### 2.1. Participants

The minimum sample size was estimated based on the total population of registered veterinarians in Spain in 2024 (N = 35,350; according to data from the INE, 2025). To estimate a mean with a standard deviation of 8 points on the ProQOL subscales (Macía et al., 2022), a confidence level of 95%, and a margin of error of ±2 points, the calculation for finite populations yielded a minimum of 61 participants. However, the requirements of the planned statistical analysis were also considered. For the confirmatory factor analysis, the calculation using G*Power (v. 3.1.9.7) indicated a minimum of 129 subjects; for the K-means cluster analysis, a minimum sample of 120 participants was estimated (116 according to one-way ANOVA); and for the ordinal regression with three dichotomous predictors, a minimum of 123 was obtained. Adopting the most conservative criterion, the minimum recommended sample size was 129 participants, which guarantees the statistical power and reliability necessary for the study objectives.

Therefore, the final sample consisted of 135 individuals who met the following inclusion criteria: (a) being active at the time of the research; (b) working in a veterinary clinic or service, maintaining direct contact with animals and their owners; and (c) not being in psychological or psychiatric treatment at that time or having been in the previous twelve months. Those who did not meet any of these criteria were excluded from the study.

In addition, the sampling error associated with the prevalence rates of burnout (2%) and secondary traumatic stress (4.3%) reported by Macía et al. (2022) in the Spanish veterinary population was estimated. For a sample of 135 subjects and a 95% confidence level, the maximum margin of error is ±3.42% (assuming *p* = 0.5), whereas the observed margin of error, based on the sample proportion, is ±2.36%. These values indicate an adequate level of statistical precision for preliminary estimates of the distribution of these phenomena in the target population, as well as for exploring their association with sociodemographic variables.

Regarding the distribution of sociodemographic characteristics in the sample (see Table 1), the proportion of women (65.9%) is very similar to that recorded nationally in the veterinary profession (69.4%; INE, 2025), so it cannot be considered a significant overrepresentation. In relation to age, professionals aged below 44 years old appear to be overrepresented (61.5% compared to 45.4% nationally), possibly due to the greater use of digital channels for the dissemination of the questionnaire or to a participation bias associated with age. Finally, 63.7% of participants lived with another person, a proportion very close to the 61.2% of the general Spanish population (CIS, 2025), which rules out any significant deviations in this variable.

### 2.2. Sampling Procedure and Data Collection

A non-probabilistic, intentional, and convenience sampling procedure was used. Participants were recruited by sending letters of invitation to various clinics and veterinary services distributed across different autonomous communities. The letter explained the objectives of the study and requested the collaboration of the entities in disseminating the link to the survey among their staff.

The questionnaire was available in electronic format during March and April 2024. Participation was voluntary, anonymous, and unpaid. Before beginning, informed consent was requested through an explicit statement detailing the objectives of the study, the confidential nature of the data, and the impossibility of withdrawing responses once submitted, given the anonymous nature of the form. In addition to the institutional channel, the survey was disseminated through professional social networks, official veterinary associations, and sectoral organizations, to ensure greater geographical and functional diversity among the participating professionals.

### 2.3. Instruments

The survey used in the study consisted of the following sections:

Sociodemographic variables: Information was collected on gender, age, and marital status/cohabitation with a partner.

Professional Quality of Life Scale: The ProQOL 5 version (Stamm, 2010) was used, which is publicly available in Spanish at the following web address: https://proqol.org. This scale consists of 30 items scored on a six-point Likert scale (0 = never; 5 = always). It assesses two main dimensions: compassion satisfaction (CS) and compassion fatigue (CF), the latter subdivided into burnout (BO) and secondary traumatic stress (STS).

The wording of some items was specifically adapted to the field of animal care by replacing the words “people”, “someone” or “those” by “animal” or “animals”.

Open comment: The survey closed with an open-ended question designed to gather impressions, feelings, or concerns related to the respondents’ professional experience. The responses obtained were analyzed using content analysis, semantic networks, and sentiment analysis techniques. This complementary qualitative analysis allowed us to identify thematic categories of emotional distress, explore their co-occurrence, and estimate associations with sociodemographic variables and CF levels, with the aim of enriching our understanding of the phenomenon based on the participants’ direct narratives.

### 2.4. Data Analysis

To address the study objectives, we designed a sequential analytical strategy integrating quantitative and qualitative techniques to characterize emotional risk profiles among veterinarians and explore their underlying determinants.

#### 2.4.1. Quantitative Analysis

Quantitative analyses were performed using BM SPSS Statistics (v. 29.0.1.0), R (v. 4.5.1.; lavaan v. 0.6, cluster v. 2.1.8.1, ordinal 2023. v. 12-4.1, MASS v. 7.3, qreg v. 6.1 packages) and JASP (v. 0.95.3.0; for AFC), establishing a significance level of *p* < 0.05. First, missing values were examined. Cases with more than 10% omissions in the main ProQOL items were excluded, while those with complete data in the key variables were retained. The pattern of missing data was evaluated using Little’s MCAR test, with no evidence of systematic bias found. Given the low volume and random nature of the omissions, imputation techniques were not applied.

The structural validity of the ProQOL was examined through a confirmatory factor analysis (CFA).

Hierarchical and non-hierarchical cluster analyses (K-means) were performed on these scores to identify distinct emotional profiles. The robustness of the segmentation was evaluated using stability indices (Jaccard), discriminant analysis, silhouette indices, and contrasts through GLM models.

The prevalence of CF was estimated from the identified profiles, distinguishing high-risk configurations (characterized by high CF and low CS) from profiles of moderate distress with high satisfaction, which facilitated clinical interpretation and informed more refined preventive recommendations.

Finally, an ordinal regression model (cumulative logit) was applied to examine the predictive value of sociodemographic variables (gender, dichotomized age, and cohabitation) on belonging to the identified emotional profiles.

#### 2.4.2. Qualitative Analyses

Complementarily, the participants’ open comments (*n* = 31) were analyzed using inductive categorical content analysis. After segmenting the responses into minimal units of meaning, they were coded into thematic categories through a process of independent double coding and consensus resolution. Interrater reliability prior to consensus was high (Cohen’s κ = 0.83; 92% agreement), indicating substantial consistency between coders. The final categories were treated as dichotomous variables (presence/absence) for each participant.

Thematic coincidence indices between pairs of categories were calculated using the Jaccard coefficient, which allowed a semantic network of complaints to be represented graphically using the ForceAtlas2 algorithm (Gephi v. 010), and networkx (Python v. 3.10), and Mind Manaer (v. 21.1.231) to enhance visual clarity and facilitate the interpretation of relationships among categories.

In addition, sentiment analysis was applied using automated lexicographic techniques (syuzhet package, R), using the NRC dictionary adapted to Spanish. Emotions were classified according to Plutchik’s model (Plutchik, 1980), differentiating between negative (anger, fear, sadness, disgust) and positive (joy, confidence, anticipation, surprise) emotions, as well as in general dimensions of affective valence.

Finally, ordinal and binary logistic regression models were estimated to explore the relationship between the number and type of verbalized complaints, sociodemographic variables, and emotional risk profiles. The total number of negative categories mentioned was used as an ordinal dependent variable, interpreted as an indicator of accumulated complaints.

## 3. Results

### 3.1. Factor Analysis of the ProQOL Scale

A confirmatory factor analysis (CFA) was conducted to test the original three-factor structure of the ProQOL (Stamm, 2010). The model showed poor fit to the data (χ^2^ (405) = 1190.34, *p* < 0.001; CFI = 0.595; TLI = 0.565; NFI = 0.498; RMSEA = 0.117; PNFI = 0.464; IFI = 0.601; AIC = 11,606.56; BIC = 11,780.88), indicating that the original three-factor solution was not supported. Based on modification indices (MIs), item loadings, and semantic redundancy, a more parsimonious model with 11 items grouped into two factors—compassion fatigue (CF) and compassion satisfaction (CS)—was retained. This model demonstrated an acceptable fit (χ^2^ (43) = 122.21, *p* < 0.001; CFI = 0.888; TLI = 0.857; RMSEA = 0.117 [90% CI: 0.093–0.141]; SRMR = 0.080), supporting its adequacy despite the slightly elevated RMSEA.

The factor loadings were adequate (≥0.45; see Table 2), and the negative covariance between CF and CS (r = −0.285, *p* = 0.012) confirms that both dimensions are opposite but related. The reliability (α = 0.87 and 0.83; total Ω = 0.92 and 0.87) and convergent validity (AVE = 0.54 and 0.53) analyses were satisfactory.

Discriminant validity was also supported, as the criterion of Fornell and Larcker (1981) was met: the square root of AVE was greater than the correlation between factors (√AVE_HR = 0.737; √AVE_CS = 0.725; r = −0.285), supporting that CF and CS are distinguishable constructs.

Finally, a model with a general factor (items loading on a general factor of emotional impact and its specific factors) showed lower fit and reliability than the two-factor correlated model. Therefore, the shared variance is better explained by the specific factors CF and CS, without a general factor dominating the structure.

### 3.2. Cluster Analysis

#### 3.2.1. Cluster Identification

For exploratory purposes, we first ran a hierarchical cluster analysis (average linkage/UPGMA; rescaled Euclidean distance). The dendrogram suggested a four-cluster solution. The cophenetic correlation was 0.662, indicating moderate fit; therefore, we refined the partition with a k-means (k = 4) seeded from the hierarchical solution.

This analysis identified four distinct profiles based on CF and CS scores:

Cluster 1 (*n* = 37; 27.4%) was characterized by above-average scores in both dimensions, suggesting intense emotional involvement.

Cluster 2 (*n* = 36; 26.7%) showed low scores on both dimensions, consistent with a profile of emotional detachment.

Cluster 3 (n = 31; 23%) showed a low level of CF and moderate CS, consistent with functional distancing.

Cluster 4 (*n* = 31; 23%) combined a high level of CF with low CS, constituting a high emotional risk profile.

**Figure 1:**
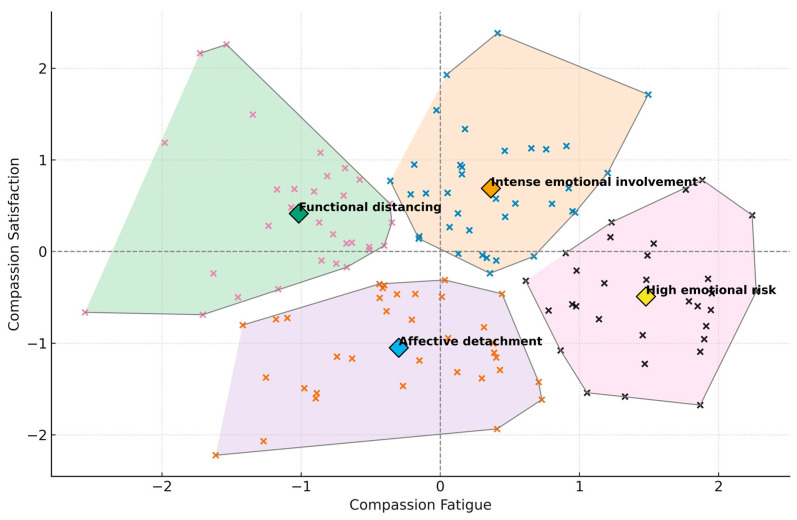
Four-cluster solution on CF and CS.

Figure 1 displays the individual factor z-scores, the spatial delimitation of each cluster and their centroids, allowing inspection of distribution and relative overlap across profiles.

#### 3.2.2. Validation and Robustness of the Clustering Solution

To examine the stability of the classification obtained, the resampling procedure with replacement (cluster boot) was applied to the K-means analysis, calculating the Jaccard index for each cluster. The results showed acceptable stability in cluster 1 (J = 0.69), but lower levels in the remaining clusters: cluster 2 (J = 0.51), cluster 3 (J = 0.34), and cluster 4 (J = 0.52), probably because of profile overlaps and the smaller cluster sizes.

**Figure 2:**
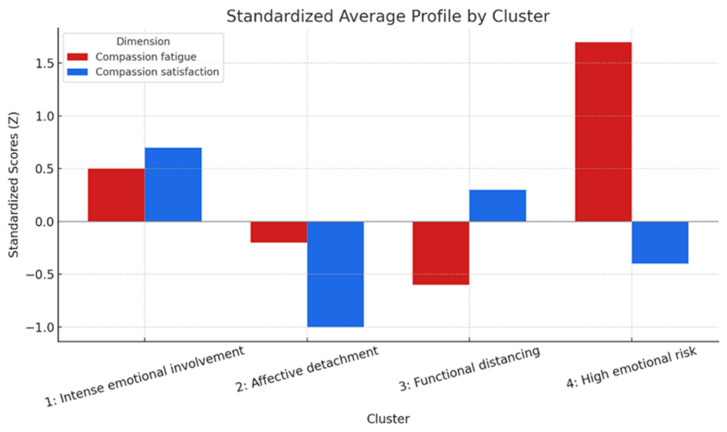
Standardized mean profile in the dimensions of fatigue and compassion satisfaction by group (K-means).

The graphical representation of the mean profiles (standardized means of each variable per cluster; see Figure 2) showed patterns consistent with the theoretical interpretation presented in the previous point.

To evaluate the quality of the four-cluster solution, the silhouette index was calculated for each case, as well as the mean value for the entire structure, which was 0.51. The mean silhouette values across clusters ranged from 0.49 (cluster 1) to 0.53 (cluster 4), indicating a reasonably well-defined configuration in which individuals were closer to members of their own cluster than to those of other clusters. None of the clusters presented negative or near-zero values, confirming the internal cohesion and interpretability of the four-group solution.

Overall, these results suggest that the four-cluster solution presents an interpretable and stable structure suitable for segmenting the emotional profiles of veterinarians. This segmentation provides a solid basis for a more detailed analysis of differences and relationships with other variables.

#### 3.2.3. Discriminant Analysis

A linear discriminant analysis was performed to determine whether the standardized scores for fatigue (ZCF) and compassion satisfaction (ZCS) could reliably differentiate between clusters. The dependent variable was membership in one of the clusters. Equal probabilities were assumed, and the intragroup covariance matrix was used.

The test of homogeneity of covariance matrices (Box’s M) was not significant (F = 1.065; *p* = 0.311), supporting the validity of the linear model under the assumption of equal covariances between clusters.

The analysis generated three discriminant functions, of which only the first reached statistical significance (Λ de Wilks = 0.250; χ^2^ (6) = 165.51; *p* < 0.001), explaining 89.3% of the total variance. The second and third functions were not significant (*p* = 0.454 and *p* = 0.963, respectively).

The first discriminant function, with a canonical correlation of 0.798, showed strong power of separation between clusters, determined mainly by HR (structural coefficient = 0.948), while HRV contributed secondarily and inversely (structural coefficient = −0.284). This pattern suggests that high levels of CF and low levels of CS characterize the profiles with the highest emotional risk.

The analysis of the centroids showed a clear progression along the first function: cluster 3, the very low risk cluster, had the most negative value (−2.531), followed by cluster 2 (−0.793), the low-moderate risk cluster, followed by cluster 1 (1.144), which represents moderate-high risk, and cluster 4 (2.18), which represents very high risk. This distribution indicates that the function effectively discriminates in the direction of increasing emotional risk.

The correct classification rate reached 88.1% in the original sample and remained high after applying cross-validation using the “leave one out” procedure (87.4%). In both cases, the classification was particularly accurate in the extreme groups: the very low-risk cluster (No. 3) was correctly classified in 96.3% of cases, and the very high-risk cluster (No. 4) in 93.5%. The intermediate groups also showed satisfactory classification rates (cluster 2: 79.4%; and cluster 1: 83.3%).

These results show that the combination of ZCF and ZCS effectively discriminates between different levels of emotional risk. CF emerges as the most powerful predictor in the discriminant structure, while CS provides a complementary nuance.

The high classification rate empirically validates the identified cluster structure and suggests that these dimensions can be used for diagnostic or screening purposes in professional populations at risk of emotional exhaustion.

#### 3.2.4. GLM Differences Between Clusters

A general linear multivariate analysis (GLM) was performed to examine whether there were significant differences in levels of compassion fatigue (CF) based on the emotional profiles identified by cluster analysis, controlling for the effect of sociodemographic variables: gender, dichotomous age, and cohabitation. The CF score was introduced as a continuous dependent variable, derived from the AFC and therefore conceptually suitable for use in GLM models.

The model results indicated a significant main effect of the clustering factor on CF, even after controlling for covariates (F (3,127) = 125.24, *p* < 0.001, partial η^2^ = 0.747), implying a very high effect size.

The sociodemographic covariates did not reach individual statistical significance, although cohabitation showed a marginal trend (*p* = 0.064, partial η^2^ = 0.027), suggesting a possible weak moderating influence on fatigue levels.

Levene’s test was significant for the HR variable (F (3,130) = 2.690, *p* = 0.049), indicating some heterogeneity of variances. However, given the relatively balanced sample size per cluster, the interpretation based on post hoc comparisons with Sidak correction was maintained.

**Figure 3:**
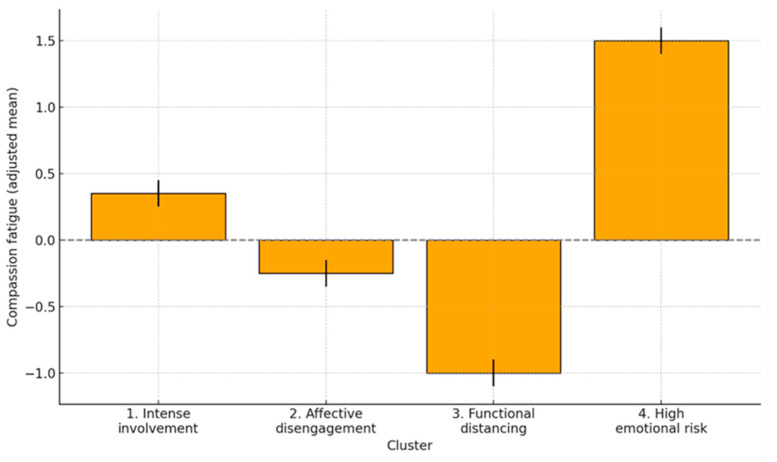
Adjusted means of compassion fatigue by group (95% CI).

The estimated marginal means (adjusted for age, sex, and partner) revealed clear differences between profiles, as shown in Table 3 and graphically represented in Figure 3, allowing for a more accurate observation of the magnitude of the differences while controlling for covariates.

All pairwise comparisons between groups were statistically significant (*p* < 0.001) with large differences between group 4 and the rest. The following table shows the adjusted mean differences in CF between groups (see Table 4).

Overall, the results confirm that differences in CF between the identified emotional profiles cannot be attributed to age, gender, or marital status. These differences remain robust even when controlling for individual variability in these covariates, reinforcing the validity of emotional typology as a differentiating criterion in fatigue risk.

### 3.3. Predictive Value of Sociodemographic Variables: Ordinal Regression Model

In order to examine the extent to which the sociodemographic variables recorded predict perceived emotional risk, an ordinal regression analysis was performed. The dependent variable was membership in one of the four clusters, which were classified hierarchically according to perceived emotional severity based on CF and CS scores, from lowest to highest: (1) functional distancing, (2) emotional detachment, (3) intense emotional involvement, and (4) high emotional risk.

The PLUM (Polymeric Universal Model) procedure was applied with a cumulative logit link function, assuming that the proportional odds hypothesis was satisfied. The predictive variables were gender (0 = male, 1 = female), dichotomized age (0 = ≤44 years, 1 = >44 years), and cohabitation (0 = no, 1 = yes). The reference category was the profile with the lowest emotional severity (functional distancing).

Initially, a complete model was estimated that included all first-, second-, and third-order interactions between the predictor variables. However, this model was not significant (χ^2^= 32.038; df = 21; *p* = 0.058), so a more parsimonious model focused on the main effects was chosen.

The final model showed an adequate fit (χ^2^ = 15.963; df = 3; *p* = 0.001), with moderate explanatory power according to the pseudo-R^2^ coefficients: Cox and Snell = 0.113, Nagelkerke = 0.125, and McFadden = 0.047. Similarly, the parallel lines test was not significant (χ^2^ = 9.707; df = 6; *p* = 0.137), confirming the validity of the probability proportionality hypothesis and justifying the use of the ordinal model.

In terms of individual effects, gender did not reach statistical significance (B = 0.169; *p* = 0.518; OR = 1.184), suggesting that there is no clear difference between men and women in the risk of belonging to more severe emotional profiles. In contrast, age and cohabitation were significantly associated with this risk. Specifically, participants over the age of 44 were more than twice as likely to belong to higher-risk profiles (B = 0.744; *p* = 0.011; OR = 2.105), as were those who lived with their partner (B = 0.662; *p* = 0.023; OR = 1.938). The model coefficients, along with their 95% confidence intervals, are presented in Table 5.

### 3.4. Qualitative Analysis of Open-Ended Comments

The open-ended comments provided by participants at the end of the questionnaire (n = 31) generated a total of 54 units of analysis, as several of them expressed more than one form of discomfort. Using inductive categorical analysis of multiple responses, these units were classified into six main thematic categories by two independent judges, who resolved their disagreements by consensus.

These categories and their relative frequencies are summarized in Table 6, which constitutes the codebook of the qualitative analysis. The most frequent themes referred to lack of professional recognition, conflicts with owners, and work overload, followed by reports of disrespectful treatment, vocational ambivalence, and isolated mentions of suicidal ideation.

Since this is a multiple-response analysis, the cumulative percentages exceed 100% (174.2%), indicating that a significant proportion of participants reported distress in more than one category. This pattern of concurrence allows for a more nuanced and richer understanding of the experiences of professional distress in the sample analyzed.

**Figure 4:**
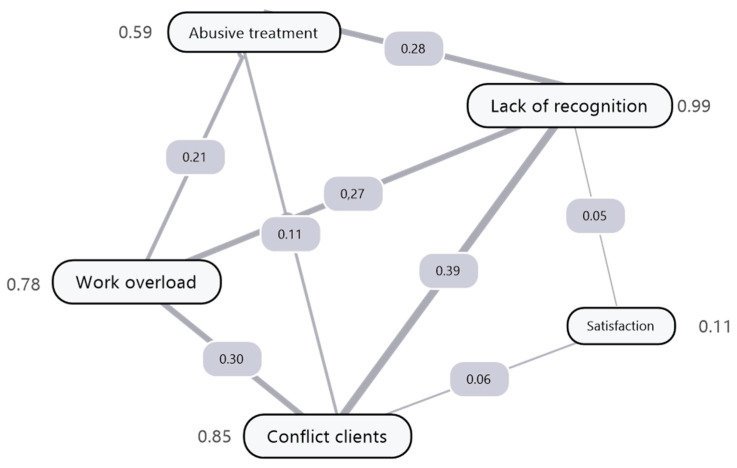
Semantic network representation of the relationships among thematic categories. The thickness of the edges represents the intensity of the association between nodes, while the numerical values correspond to the normalized relationship coefficients obtained from the co-occurrence analysis.

To explore the relationships between these categories, a semantic network of co-occurrences was constructed using the Jaccard similarity coefficient as a measure of thematic proximity. Figure 4 shows this network, in which each node represents a category of discomfort and each edge indicates the strength of overlap between two categories, based on the value of the Jaccard coefficient. The higher this value, the more frequently participants mention both categories together.

The numerical values next to each node indicate the weighted degree of its frequency; that is, the sum of the similarity coefficients with the other categories in the network. This measure allows us to identify which categories play a more central or articulating role in the discourse of discomfort. In this sense, lack of recognition stands out as the most central node (weighted degree = 0.992), followed by conflicts with owners (0.852), work overload (0.787), and humiliating treatment (0.597). These four categories form a dense relational core that predominantly structures the narratives of discontent.

In contrast, vocational satisfaction, with a weighted score of 0.106, appears in a peripheral position, weakly connected to the rest of the network, suggesting that positive or resilient content is rarely expressed alongside explicit forms of discontent. The category of suicidal ideation, although not shown in the figure due to its low connectivity, did not occur alongside any other label, reinforcing its isolated nature and suggesting a specific vulnerability profile.

Sentiment analysis, performed using the NRC Emotion Lexicon adapted to Spanish, confirmed the predominance of negative emotions in the collected discourses. The most frequent were anger, sadness, and disgust, followed by fear. In contrast, positive emotions such as joy, confidence, and anticipation were rarely mentioned. This affective pattern reinforces the interpretation of the comments as expressions of accumulated distress, professional discontent, and emotional exhaustion.

From an explanatory perspective, the relationship between sociodemographic variables and the degree of verbalized distress was explored. The ordinal regression model showed that age was a significant predictor of the number of categories of distress expressed (χ^2^ = 8.235; *p* = 0.004), with a higher accumulation in professionals aged 44 or younger (B = 2.485; 95% CI = 0.788–4.181). Living with a partner had a marginally significant effect (χ^2^ = 3.844; *p* = 0.050), with a negative association (B = −1.585; 95% CI = −3.169 to −0.001), suggesting that those who do not live with a partner tend to verbalize a greater number of complaints. Gender was not found to be a significant predictor (χ^2^ = 0.472; *p* = 0.492).

Regarding the binary logistic regression model to predict the group with high CF, the variable «lack of recognition» emerged as a marginal predictor (B = −22.472; *p* = 0.998; OR = 0), although this estimate lacks numerical stability and should be interpreted with caution. The model showed a modest fit (Nagelkerke’s R^2^ = 0.166) and a correct classification rate of 67.7%.

Finally, multinomial logistic regression revealed that the set of distress categories allows for significant discrimination between the different clusters (χ^2^ [18] = 43.57; *p* < 0.001; Nagelkerke’s R^2^ = 0.820).

## 4. Discussion

### 4.1. Confirmation of the Structural Validity of the ProQOL in the Veterinary Population

Although psychometric validation was not the main objective, verifying the structural adequacy of the ProQOL was essential for interpreting subsequent analyses. In our sample of Spanish veterinarians, the original three-factor model (CS, BO, and STS; Stamm, 2010) did not fit adequately. Consistent with previous evidence questioning the discriminant validity between BO and STS in healthcare contexts (Coetzee & Klopper, 2010), the two-factor correlated model—compassion fatigue (CF), integrating BO and STS items, and compassion satisfaction (CS)—provided a better fit.

This solution, consistent with recent findings (Rohlf et al., 2022), supports the view of CF as a unified form of affective exhaustion rather than two separate constructs. The negative correlation between CF and CS confirms that both reflect opposite, though not mutually exclusive, poles of the emotional impact of work (Figley, 2002; Wolf et al., 2024).

In the veterinary context, characterized by the professional–animal–owner triadic relationship and the tension between animal welfare and client expectations, this two-dimensional structure appears parsimonious and conceptually coherent (Hill et al., 2020; Kogan & Rishniw, 2023; Kogan et al., 2020). It provides a robust framework for identifying risk profiles and supports the use of ProQOL in studies of occupational quality of life among veterinarians.

### 4.2. Emotional Profiles in Veterinarians: A Typological Approach

Rather than focusing on mean differences, the four-cluster typology clarifies how veterinarians combine distress and gratification in practice. The two dysfunctional configurations—High emotional risk and Affective detachment—map onto distinct failure modes of emotional regulation: the former reflects strain under sustained demands with depleted recovery, whereas the latter suggests protective blunting that can drift into demotivation or depersonalization. By contrast, Intense emotional involvement and Functional distancing appear adaptive pathways that balance engagement and protection, the former via purpose/meaning (high CS despite early CF signals) and the latter via regulated investment (low CF with moderate CS).

From a prevention standpoint, this typology is actionable. It supports profile-tailored interventions: monitoring load and recovery and strengthening restorative practices for High emotional risk; addressing motivational climate and reconnecting with professional values for Affective detachment; and consolidating protective routines (boundary setting, recovery micro-breaks, peer support) for the two functional profiles to avoid drift toward risk. Importantly, profiles also offer a better segmentation lever than broad sociodemographic categories for targeting resources in busy settings.

Methodologically, the convergence of the clustering with discriminant and GLM checks speaks to construct coherence rather than sample idiosyncrasy, without altering the substantive message: compassion fatigue drives risk separation, while compassion satisfaction refines adaptive distinctions. This aligns with calls for personalized, integrative prevention in caregiver stress (e.g., Perret et al., 2020; Schlanser et al., 2021).

Two caveats merit attention. First, profiles are states, not traits; longitudinal designs should examine transitions between configurations (e.g., from intense involvement to high risk under cumulative demands). Second, as profiles were derived from two dimensions, adding contextual and organizational markers (workload, control, recognition) may sharpen boundaries and enhance implementation utility.

### 4.3. Empirical Estimation of the Prevalence of Compassion Fatigue

Based on the latent profiles identified, approximately 23% of Spanish clinical veterinarians can be considered at high emotional risk (high CF and low CS). This estimate falls within the mid-range of values reported in the literature and aligns with moderate figures found among similar caregiving professionals. In contrast, it differs from the lower prevalence obtained using more restrictive instruments or cut-off points (e.g., Macía et al., 2022) and from the markedly higher rates observed in other animal-care contexts (e.g., González-Ramírez & Landero-Hernández, 2025). Such discrepancies likely reflect variations in measurement instruments (ProQOL vs. MBI), cut-off criteria, sampling strategies, and work settings (clinical vs. shelter). From a practical standpoint, this 23% underscores the importance of targeted interventions for high-risk professionals and of regular monitoring to prevent chronic emotional deterioration.

### 4.4. Predictive Value of Sociodemographic Variables in Perceived Emotional Risk

The cumulative ordinal logit model revealed that certain sociodemographic variables—gender, age, and cohabitation with a partner—differentially predicted perceived emotional risk, as defined by the profiles identified through cluster analysis. The model showed a statistically significant fit and met the proportional odds assumption.

Gender did not emerge as a significant predictor, which contrasts with studies reporting higher BO and STS levels among women (Best et al., 2020; Wolf et al., 2024; Young et al., 2024). However, these differences often disappear when multivariate controls are applied (Perret et al., 2020; Rohlf et al., 2022; Schlanser et al., 2021), suggesting that the greater emotional burden attributed to women may be mediated by contextual or psychological factors, such as coping styles or socially assigned caregiving roles.

In contrast, being over 44 years of age and living with a partner were associated with a higher probability of belonging to high-risk profiles. This pattern diverges from findings that indicate greater vulnerability among younger professionals (Best et al., 2020; Dow et al., 2019) and from the commonly held view of spousal support as a protective factor (Dow et al., 2019; Goñi-Balentziaga & Azkona, 2024).

At first glance, the qualitative findings seem to contradict the regression model, since younger professionals and those living with partners more frequently verbalized distress, including frustration, overload, and lack of recognition. However, this apparent discrepancy may reflect complementary rather than opposing patterns. Qualitative analysis is based on a self-selected subsample more likely to express discomfort, whereas quantitative models integrate configurations of fatigue and satisfaction, in which verbalization and risk profile do not necessarily coincide. Younger individuals may exhibit more reactive and visible distress, while older professionals accumulate sustained but less explicit fatigue. Similarly, cohabitation may serve as either a protective or amplifying factor, depending on the balance between work and personal life (Reif-Stice et al., 2023).

These findings caution against simplistic interpretations of sociodemographic effects and reinforce the value of mixed-method approaches for capturing both narrative expressions and latent risk patterns (cf. Andrukonis et al., 2020; Rohlf et al., 2022; Goñi-Balentziaga & Azkona, 2024). Overall, they underscore the importance of comprehensive, interdisciplinary prevention strategies tailored to emotional profiles and career trajectories, promoting a more sustainable and emotionally healthy professional practice.

### 4.5. Theoretical and Practical Contributions of the Study

This study strengthens the conceptual basis of compassion fatigue (CF) in veterinary medicine from both theoretical and applied perspectives. The better fit of a two-factor ProQOL structure—compassion fatigue and compassion satisfaction—over the general or three-factor models indicates that shared variance is more coherently organized into specific dimensions, consistent with recent evidence questioning the three-factor validity in clinical contexts (Rohlf et al., 2022).

From a practical standpoint, the four-profile segmentation (functional distancing, emotional detachment, intense emotional involvement, and high risk) provides a more ecological and nuanced classification than conventional cut-off criteria, capturing the ambivalent interplay between exhaustion and gratification that characterizes veterinary practice.

Quantitative and qualitative findings converge in showing that older age and cohabitation with a partner are linked to a higher probability of belonging to high-risk profiles, whereas younger professionals without a partner more often verbalize distress related to overload, owner conflicts, and lack of recognition. Rather than contradictory, these results reflect two complementary modes of distress expression, one explicit and the other cumulative, consistent with cumulative burden models. Accordingly, the design of profile-specific preventive strategies focused on the most salient stressors is warranted, integrating subjective experience with latent configurations for a more comprehensive understanding and intervention.

### 4.6. Study Limitations and Methodological Considerations

Despite the robustness of the analyses, this study has several limitations that should be considered when interpreting the results. First, its cross-sectional design prevents the establishment of causal relationships between the variables studied, limiting the possibility of identifying temporal trajectories between exposure, fatigue, and compassion satisfaction.

Second, although the sample was diverse in age, gender, and life circumstances, its size (N = 135) may have reduced statistical power in some analyses—particularly in the ordinal regression—as reflected in the pseudo-R^2^ values obtained. In the qualitative component, the number of participants who responded to the open-ended question was limited (*n* = 31), which constrains the diversity of the discourse analyzed. The brevity of some responses also hindered a more detailed coding of experiential nuances. Nevertheless, these comments added interpretative depth by illustrating forms of distress that enrich the understanding of latent emotional profiles.

Another relevant limitation concerns the self-selected nature of the online sampling procedure. Although appropriate for reaching a geographically dispersed population, it may have introduced participation bias by attracting individuals especially sensitive to or affected by the topic.

Finally, although the ProQOL items were adapted to the animal-care context, these modifications were not subjected to a systematic validation process, which could partially affect the content validity of the instrument.

## 5. Conclusions

Despite the limitations noted, this study contributes significantly to understanding the emotional impact of veterinary clinical work by validating a two-dimensional structure of the ProQOL adapted to this context and proposing an empirical segmentation of professionals based on their emotional profiles. Through the typological approach used, the limitations of models based on fixed cut-off points have been overcome, providing a richer and more contextualized view of the phenomenon of compassion fatigue.

The results obtained show that more than half of the sample presents clinical indicators of emotional exhaustion, either in the form of high risk or intense involvement coexisting with gratification. Likewise, it has been demonstrated that sociodemographic variables such as age and cohabitation with a partner may be associated with greater vulnerability, which challenges some traditional conceptions about protective factors in the workplace.

From an applied perspective, these findings can guide the design of preventive programs tailored to these risk profiles, as well as promote organizational policies that are sensitive to the life trajectories and relational contexts of professionals. In addition, the study lays the foundation for future longitudinal research that analyzes the evolution of these profiles over time and integrates psychological and organizational variables that modulate emotional risk.

Overall, the study reaffirms the need to address the well-being of veterinary staff from a comprehensive approach that considers both the suffering observed and the sources of professional gratification, without dissociating the emotional from the structural or the personal from the relational.

Finally, the qualitative analysis of the comments has identified areas for intervention that complement the structural data, highlighting the importance of professional recognition, relationships with animal guardians, and the perception of overload. These elements, which are difficult to capture using closed scales, add value to the design of programs that are more sensitive to the subjective experiences of professionals.

## Figures and Tables

**Table 1:** Demographic characteristics of the sample.

Variable	Categories	Frequency	Percentage
Gender	Females	89	65.9
	Males	45	33.3
	Prefer not to answer	1	0.8
Age	25–34	40	29.6
	35–44	43	31.9
	45–54	35	25.9
	Over 54	17	12.6
Marital status	Married/in a relationship	86	63.7
	Single	41	30.4
	Divorced	8	5.9

**Table 2:** Content and standardized factor loadings.

Factors	Items (Original Scale Number)	Loadings
Compassion fatigue	I lose sleep because of the traumatic experiences of the animals I have helped (8).	0.873
	I believe that I have been negatively affected by the traumatic experiences of the animals I have helped (9).	0.873
	Because of my profession, I feel like I am at my limit in several areas (11).	0.727
	I feel as if I am the one experiencing the trauma of an animal I have helped (14).	0.715
	Because of my job, I feel exhausted (19).	0.594
	I find it difficult to separate my personal life from my professional life (7).	0.559
Compassion satisfaction	My work makes me feel satisfied (18).	0.873
	I am happy that I chose to do this job (30).	0.818
	I am satisfied with how I am able to keep up to date with veterinary care techniques and procedures (16).	0.692
	I have positive thoughts about the animals I have helped and how I have been able to help them (20).	0.658
	I consider myself to be a good professional (27).	0.450

**Table 3:** Raw mean, adjusted mean, and confidence intervals (95% CI) for compassion fatigue according to emotional profile ^1^.

Cluster	N	Mean CF (Raw)	HRT (Raw)	Adjusted Mean HR	SE Adjusted CF	95% CI (Lower)	95% CI (Upper)
1. Intense emotional involvement	37	0.363	0.426	0.352	0.85	0.184	0.52
2. Emotional detachment	36	−0.299	0.636	−0.293	0	−0.467	−0.119
3. Functional distance	30	−0.103	0.524	−1.035	0.95	−1.223	−0.847
4. High emotional risk	31	1.477	0.457	1.489	0.95	1.301	1.677

^1^ Adjusted means were calculated controlling for gender, dichotomous age and cohabitation with a partner.

**Table 4:** Adjusted mean differences between groups in compassion fatigue.

Comparison	Difference in Means	95% CI
Cluster 1 vs. 2	0.645	(0.316–0.974)
Cluster 1 vs. 3	1.387	(1.048–1.726)
Cluster 1 vs. 4	−1.137	(−1.478–−0.796)
Cluster 2 vs. 3	0	(0.395–1.089)
Cluster 2 vs. 4	−1.782	(−2.134–−1.429)
Cluster 3 vs. 4	−2.524	(−2.886–−2.162)

**Table 5:** Results of the ordinal regression model: sociodemographic variables as predictors of emotional risk level ^1^.

Predictor	B	Standard Error	*p*	OR (Exp(B))	95% CI OR (Lower Limit–Upper Limit)
Sex (female)	0.169	0.263	0.518	1.184	0.707
Age > 44 years	0.744	0.293	0.011	2.105	1.192–3.716
Living with a partner	0.662	0.292	0.020	1.93	1.098

^1^ Dependent variable: belonging to emotional risk profiles derived from cluster analysis. Reference category: least severe profile (functional distancing).

**Table 6:** Thematic categories ordered by frequency (n, % of responses, and % of cases), operational definitions, and illustrative examples derived from the qualitative analysis of open-ended comments (n = 31; 54 meaning units).

Thematic Category	Operational Definition	Illustrative Example	n (Units)	% of Responses	% of Cases
Lack of professional and social recognition	Comments reflecting the perception of undervaluation of veterinary work, both economically and socially.	“The low social appreciation and high demands of our job make it hard to keep going.” (Subj. 91)	17	31.5	54.8
Conflicts with animal owners	Mentions of tension, excessive demands, or aggressive behavior from animal owners that interfere with professional practice.	“The problem in this job isn’t the animals, it’s their owners.” (Subj. 131)	15	27.8	48.4
Work overload and lack of work–life balance	References to excessive workload, lack of rest, or difficulty reconciling personal and professional life, particularly among self-employed veterinarians.	“If you’re committed to this profession, you stop being the owner of your life—especially if you’re your own boss.” (Subj. 43)	11	20.4	35.5
Abusive or over-demanding institutional environment	Descriptions of abuse, excessive pressure, or inadequate treatment from supervisors, colleagues, or clients.	“Being mistreated or verbally abused by a superior (insults, excessive demands…).” (Subj. 53)	6	11.1	19.4
Vocational satisfaction and ambivalence	Expressions of pride or fulfillment in the profession, coexisting with feelings of exhaustion or fatigue.	“This is the best job in the world, though it’s exhausting.” (Subj. 93)	4	7.4	12.9
Psychosocial risk and suicidal ideation	Comments explicitly referring to suicidal thoughts or severe psychological distress related to work.	“I have had suicidal thoughts.” (Subj. 132)	1	1.9	3.2

## Data Availability

The data from this research project will be available at idus: Research Repository of the University of Seville (https://idus.us.es).

## Abbreviations

The following abbreviations are used in this manuscript:

CF	Compassion fatigue
CS	Compassion satisfaction
BO	Burnout
STS	Secondary traumatic stress
CFA	Confirmatory factor analysis
ProQOL	Professional quality of life scale
AVE	Average Variance Extracted
UPGMA	Unweighted Pair Group Method with Arithmetic Mean.
